# A scheme to evaluate structural alerts to predict toxicity – Assessing confidence by characterising uncertainties

**DOI:** 10.1016/j.yrtph.2022.105249

**Published:** 2022-11

**Authors:** Mark T.D. Cronin, Franklin J. Bauer, Mark Bonnell, Bruno Campos, David J. Ebbrell, James W. Firman, Steve Gutsell, Geoff Hodges, Grace Patlewicz, Maria Sapounidou, Nicoleta Spînu, Paul C. Thomas, Andrew P. Worth

**Affiliations:** a School of Pharmacy and Biomolecular Sciences, Liverpool John Moores University, Byrom Street, Liverpool, L3 3AF, UK; b KREATiS SAS, 23 rue du Creuzat, ZAC de St-Hubert, 38080, L'Isle d’Abeau, France; c Science and Risk Assessment Directorate, Environment & Climate Change Canada, 351 St. Joseph Blvd, Gatineau, Quebec, K1A 0H3, Canada; d Safety and Environmental Assurance Centre, Unilever, Colworth Science Park, Bedfordshire, MK44 1LQ, UK; e Center for Computational Toxicology and Exposure (CCTE), US Environmental Protection Agency, 109 TW Alexander Dr, RTP, NC, 27709, USA; f European Commission, Joint Research Centre (JRC), Ispra, Italy

**Keywords:** Structural alert, Structure-activity relationship, Toxicity prediction, Confidence, Uncertainty, Evaluation scheme, Use case, Computational toxicology, AChE, acetylcholinesterase, AOP, Adverse Outcome Pathway, EFSA, European Food Safety Authority, HPV, High Production Volume, KE, Key Event, MIE, Molecular Initiating Event, OECD, Organisation for Economic Cooperation and Development, QMRF, QSAR Model Reporting Format, QPRF, QSAR Prediction Report Format, QSAR, quantitative structure-activity relationship, RAAF, Read-Across Assessment Framework, SAR, structure-activity relationship

## Abstract

Structure-activity relationships (SARs) in toxicology have enabled the formation of structural rules which, when coded as structural alerts, are essential tools in *in silico* toxicology. Whilst other *in silico* methods have approaches for their evaluation, there is no formal process to assess the confidence that may be associated with a structural alert. This investigation proposes twelve criteria to assess the uncertainty associated with structural alerts, allowing for an assessment of confidence. The criteria are based around the stated purpose, description of the chemistry, toxicology and mechanism, performance and coverage, as well as corroborating and supporting evidence of the alert. Alerts can be given a confidence assessment and score, enabling the identification of areas where more information may be beneficial. The scheme to evaluate structural alerts was placed in the context of various use cases for industrial and regulatory applications. The analysis of alerts, and consideration of the evaluation scheme, identifies the different characteristics an alert may have, such as being highly specific or generic. These characteristics may determine when an alert can be used for specific uses such as identification of analogues for read-across or hazard identification.

## Highlights

• Structural alerts are useful tools for predictive toxicology.
• 12 criteria to evaluate structural alerts have been identified.
• A strategy to determine confidence of structural alerts is presented.
• Different use cases require different characteristics of structural alerts.
• A Scheme to Evaluate Structural Alerts to Predict Toxicity – Assessing Confidence By Characterising Uncertainties.

## 1 Introduction

The concept of the structure-activity relationship (SAR) is fundamental to predictive toxicology (Cronin and Yoon, 2019). As such, SARs have found widespread use in toxicology, risk assessment and other regulatory applications with a particular resurgence of interest with the increasing desire to consider chemical safety without the use of animals (Worth, 2020). Key to enabling SARs as useable *in silico* tools for these applications is the development of structural rules which can then be coded computationally so that they may be applied to identify potential hazard in new molecules (Madden et al., 2020; Cronin et al., 2022). The term “structural alert” is assumed in this paper to represent a fragment or substructure within a molecule that is hypothesised to be responsible for a biological activity from a structural rule. Such a fragment is derived from SAR-based structural rules and may be associated with other structural information such as that relating to substitution patterns or parent structures.

Structural alerts can represent the chemistry which is associated with, for instance, an interaction such as a molecular initiating event (MIE) or key event (KE) in an Adverse Outcome Pathway (AOP) (Allen et al., 2018), an adverse effect (i.e., toxicity that can be observed at an organism or population level) (Siramshetty et al., 2018) or related to a regulatory endpoint (Valsecchi et al., 2019) or indicator of significant toxicity (e.g., as part of the Cramer et al. (1978) Decision Tree). The understanding that chemical properties were responsible for toxicological events was well established at the turn of the twentieth century (e.g. Meyer, 1901; Overton, 1901) along with the concept that specific chemical structures could be associated with toxicity (Landsteiner and Jacobs, 1935). The first use of the term “structural alert” is accredited to Ashby (1985) with regard to defining the structural basis of carcinogenicity, a concept that went on to define a series of alerts for genotoxic carcinogenicity (Ashby and Tennant, 1988). Since that time, alerts have been developed in many areas of toxicology for human health and environmental endpoints. The history and use of alerts in toxicology has been well reviewed recently (Cronin and Yoon, 2019; Yang et al., 2020) and a large compilation of alerts is freely available through the OCHEM website (https://ochem.eu; Sushko et al., 2011, 2012).

There are a number of ways of developing the SAR which forms the basis of structural alerts and these are summarised in Table 1, along with their characteristics and strengths and weaknesses. No method is exclusive and, in terms of understanding their use better, no analysis has been performed to determine if or when a particular method may be appropriate. From the outset, it is acknowledged that “expert knowledge” is a subjective term with no clear criteria to define it. In terms of the use of the term “expert knowledge” in this study, it is assumed that the expert would have some training or appreciation of toxicology in the context of hazard identification and be familiar with relevant data for the chemical(s) and endpoint in question.

**Table 1:** Summary of approaches to derive structure-activity relationships, and ultimately structural alerts, for predictive toxicology.

Method to derive the structural alert	Description	Characteristics in terms of data for the SAR, methodology and mechanistic understanding	Strengths	Weaknesses	Illustrative example
Expert Knowledge Based on Toxicological Data	Derived from the knowledge of toxicologists who have experience in assessing the data associated with toxicological properties of a series of chemicals	Data: small number of toxicological data on which to base a hypothesis Methodology: Expert judgement and opinion Mechanistic: Presumed high, through precise mechanistic definition may not be possible	Derived from a knowledge based on experimental data, supported by mechanistic information	Slow to develop, no performance statistics; may be a misinterpretation from flawed data or a subjective interpretation of data	Ashby and Tennant (1988) who compiled knowledge on genotoxic carcinogens
Expert Knowledge Based on Mechanistic Understanding	Derived from expert knowledge following (non-statistical) analysis of a data set of chemicals using a mechanistic hypothesis	Data: large number of mechanistic data Methodology: Expert judgement and opinion Mechanistic: Clear mechanistic hypothesis	Based on expert knowledge (preferably from multiple sources) and potentially creating a broad set of alerts, supported by data or mechanistic understanding. Can be extended broadly without extensive toxicological data.	Labour intensive to develop and requires expert knowledge across a complete mechanism of action or dataset	Enoch and Cronin (2010) and Enoch et al. (2011) who derived alerts for DNA and protein binding respectively on the basis of electrophilic chemistry; Bauer et al. (2018) who derived a decision tree on six classes of mechanisms of action, termed MechoA
Data-Driven Approaches	Use of statistical analyses to determine fragments associated with a particular toxicity	Data: Large data sets required for analysis Methodology: data mining and machine learning of toxicological data Mechanistic: Not possible unless assigned after alert development	A rapid method, with readily available performance statistics. The data on which the alerts are derived from are available	Requirement for large data sets to achieve significant results. Prone to limited validation (usually restricted to curation). Difficult to assign mechanistic knowledge or validity to the alerts derived as they may be in an uninterpretable “black box” form. Often the fragments are overlapping and require rationalisation	Wedlake et al. (2020) used a Bayesian approach to develop alerts for *in vitro* data related to MIEs; Claesson and Minidis (2018) to develop alerts for reactive metabolite formation; Cui et al. (2019) alerts from fingerprints for drug-induced rhabdomyolysis
Chemotype Enrichment	Use of statistical analysis to determine which structural fragments may be significantly associated with a toxicity or effect	Data: Large data sets Methodology: Data mining of high throughput data Mechanistic: Driven by the mechanistic hypothesis of the data	Rapid to apply. Provides a statistical outcome to demonstrate the strength of relationship between the activity and structure. Use of readily available alerts.	Currently limited by the need for relatively large data sets and the fragments already available	Wang et al. (2019, 2021) investigated ToxCast endpoints using ToxPrint Chemotypes
Hybrid Approaches Combining Statistical Analysis and Expert Analysis	The purpose here is to use statistical analysis (such as clustering approaches) to find groups within data to be used as leads for expert analysis. This will not produce a comprehensive set of alerts but may find SARs (which can be optimised) that would not be obtained by expert knowledge alone.	Data: Many toxicological data Methodology: Clustering of data following by expert judgement and opinion Mechanistic: No mechanistic understanding unless applied after alert development	A rapid approach to derive knowledge/hypotheses. Supported by data and mechanistic understanding	Evaluating the hypotheses from data mining can be slow and requires expert knowledge.	Hewitt et al. (2013) who applied expert knowledge to the results of cluster analyses on a database of hepatotoxicity data to derive useable alerts for liver toxicity. Wang et al. (2019) used a ToxPrint chemotype enrichment analysis to identify >20 distinct chemical substructural features significantly enriched for sodium-iodide symporter inhibition.

As well as the description of methods to develop structural alerts in Table 1, other characteristics of alerts could be considered to improve their use including their definition, underlying data source(s), potential domain, mechanistic relevance, coverage and performance. Whilst these are likely to be crucial for the successful use of structural alerts, they are seldom defined, although several recent studies have demonstrated that careful development of alerts can improve performance and relevance (Amberg et al., 2019; Benigni, 2021; Kalgutkar, 2020; Kalgutkar and Driscoll, 2020). In addition, the different uses of structural alerts e.g., for hazard assessment, grouping and read-across, screening etc. have not been fully described. As such, a better understanding of the properties, specifically the strengths and weaknesses, of alerts should increase confidence in their application and hence improve opportunities for acceptance, especially for regulatory purposes.

Despite the extensive development and use of structural alerts, their importance, and reliance on them in many use cases, no standardised agreed means of describing them and assessing their utility in terms of their reliability and robustness has been developed. This is in contrast with related approaches where assessment formats have been put in place, such as read-across (e.g., the Read-Across Assessment Framework (RAAF) (ECHA, 2017)) and quantitative structure-activity relationships (QSARs) (e.g. the Organisation for Economic Cooperation and Development (OECD) Principles for the Validation of QSARs (OECD, 2007), QSAR Model Reporting Format (QMRF), QSAR Prediction Report Format (QPRF) (Worth, 2010)). The lack of an agreed approach has potentially reduced confidence in the application of SARs. As such a means of evaluating structural alerts would enable confidence to be assigned to them, ensure their optimal usage and enhance their acceptability.

One means of understanding confidence in computational toxicology tools has been through the characterisation and definition of uncertainty. For example, Schultz et al. (2019) have defined the uncertainties associated with read-across and Cronin et al. (2019) have detailed areas of uncertainty, variability and bias of QSARs for toxicity prediction. The purpose of these analyses was not to conclude that a particular approach should, or should not, be used, but to assist in the validation process, identify aspects of a model that may be associated with significant levels of uncertainty and determine the overall confidence that may be assigned to a model. This approach to understanding uncertainty provides the opportunity to determine the type and level of confidence required for a predictive toxicology approach to be “fit-for-purpose” (Belfield et al., 2021).

One of the most recognised set of criteria in health sciences and toxicology to define confidence that may be associated with evidence to support a conclusion, i.e., causation, are the Bradford Hill criteria (Hill, 1965). These were adapted by Meek et al. (2014), amongst others, to assist in a weight of evidence framework for mode of toxicological action which are closely aligned to the issue of evaluating structural alerts. The revised criteria included assessment of biological concordance, essentiality of KEs, concordance of empirical observations among KEs, consistency and analogy. Whilst these adapted Bradford Hill criteria cannot be mapped directly for the assessment of structural alerts, they provide a starting point e.g., assessment of mechanisms, underlying evidence and definition. Likewise, there is as yet no agreement of the level of quantification of uncertainty that can, or should, be applied. Schultz et al. (2019) reviewed this topic as regards to read-across and concluded at the current time a simple “high, moderate, low” scheme was the most practical. It is also noted that, with regard to AOPs, more quantitative schemes have been proposed with six (Collier et al., 2016) and seven levels of “evidence” respectively (Patlewicz et al., 2013; Becker et al., 2017). Indeed, a “scientific confidence framework” has been developed by Patlewicz et al. (2015) to support the use of AOPs for regulatory purposes. This formalises a number of criteria (seven in total) that were developed by Patlewicz et al. (2013) based on analogous assessment schemes for biomarkers and QSAR. These, and other, studies demonstrate that confidence in the use of strategies for using non-animal data can be assessed in a meaningful manner to support their use. The acceptable level of uncertainty for a particular purpose, e.g. a regulatory decision, remains difficult to ascertain and is likely to be context dependent.

Given the lack of a defined set of criteria to assess structural alerts for toxicity, the aim of this investigation was to develop a scheme for their critical evaluation. Specifically, we aimed to determine how criteria for describing the confidence in structural alerts for the prediction of toxicity could be developed based on the assessment of the uncertainties of the alerts. Reference was made to adapted Bradford Hill criteria (i.e., to assess the likelihood of causation) and other schemes for computational toxicology, with the objective of assessing and numerically scoring the overall confidence that may be placed in an alert. Further, use cases for structural alerts were reviewed with the objective of determining the characteristics of alerts that may be required for certain applications in predictive toxicology.

## 2 Methods

### 2.1 Development of criteria to define the uncertainty associated with structural alerts for toxicity prediction

A set of criteria was created to define the properties of, and uncertainty associated with, structural alerts for toxicity prediction. This task was performed by the authors using expert analysis to address particular aspects of structural alerts, in part with reference to the adapted Bradford Hill criteria, which can be summarised as follows:

- Description and definition of the domain of the structural alert
- Evidence of causality e.g., mechanisms of action
- Concordance and consistency of biology e.g., supporting data
- Performance of the structural alert

In order to make the criteria useable for the evaluation of structural alerts, the broad themes stated above were defined by a larger number of definable criteria deemed practical for the description of the uncertainties of a structural alert.

### 2.2 Provisional scheme for assigning a confidence score to a structural alert

Following definition of the criteria for the uncertainty associated with a structural alert, each was categorised with definitions for low, moderate and high uncertainty to make it into a practical and workable scheme. Should any particular criterion be irrelevant to the alert, then this would be defined “not applicable”.

In order to provide the possibility of creating an overall score, individual criteria were ranked according to their potential importance when using a structural alert. The ranking was performed semi-quantitatively and undertaken using expert opinion and interpretation.

### 2.3 Assessment of use cases for structural alerts

The use cases for structural alerts to predict toxicity were scoped, representing, in particular, both regulatory use and application within industry. Specifically, use cases were sought for different applications of structural alerts with the overall aim of predicting toxicity. For each use case the desirable characteristics of an alert were defined. The desirable characteristics were based around the criteria for definition of uncertainties and were defined as low, moderate or high. The aim of this exercise was to define and identify the types of structural alerts that are most suited for a particular use case, such that these properties could be defined by the developer/user as a means to demonstrate the applicability of an alert, group of alerts or *in silico* profiler.

## 3 Results and discussion

This study aimed to develop a scheme to evaluate the uncertainty associated with structural alerts for the prediction of toxicity such that confidence in their use could be assigned. In order to develop such a scheme, cognisance was taken of a number of approaches starting with the definition of uncertainty as provided by European Food Safety Authority (EFSA) which defined uncertainty with regard to toxicological assessment as “*all types of limitations in available knowledge that affect the range and probability of possible answers to an assessment question*” (EFSA, 2018). The EFSA Guidance is based around identifying, assessing, describing and, in some cases, quantifying uncertainty and it is this definition that was applied by Cronin et al. (2019) to defining the uncertainty and other properties of QSAR models.

### 3.1 Uncertainty assessment criteria for structural alerts

The assessment of criteria relating to uncertainty was performed with the intention of providing a scheme that would assist in the evaluation of structural alerts and to determine the types of uncertainty that may be acceptable for defined scenarios. The development of criteria focused on the definition and domain(s) (in terms of the biology/toxicology predicted, chemical structure and properties, requirement for metabolic activation etc) of an alert, its mechanistic relevance, performance and the level of evidence supporting the alert. In total, twelve assessable criteria were identified that covered the main aspects of uncertainty of a structural alert, these are described in detail and with their relevance to uncertainty in Table 2.

**Table 2:** Definitions and relevance to uncertainty of the properties relating to structural alerts.

Criteria	Definition and Relevance to Uncertainty
Purpose	The purpose, or potential use, of the structure alert with regard to regulatory assessment, product development etc. and will usually be stated by the user. For low uncertainty the stated use should be clear and unambiguous e.g., for hazard identification relating to toxicity prediction or to facilitate grouping and read-across. The characteristics of the alert should be appropriate for use.
Structural Description	The functional group, or other chemical substructure, that is defined as the structural alert is unambiguously described including any modulating factors and the local molecular environment e.g., substitution patterns on a ring, branching or unsaturation on an alkyl chain etc. Clear and unambiguous definition will enable transparency and documentation.
Property Domain	The domain of the alert defined in terms of relevant physico-chemical properties (e.g., solubility, volatility), molecular descriptors (e.g. 2D, 3D properties such as dimensions), molecular properties (e.g. toxicokinetics (e.g. clearance) and any other relevant property. It is assumed that the domain of the alert will be defined on the training set, if available.
Toxicity or Relationship to Adversity	The definition of the toxicological effect that is elicited, or the adverse effect that may be related to a MIE or KE in an AOP that is associated with the structural alert. This will provide clear indication of the use of the structural alert.
Species Specificity	The structural alert is associated with effects to a particular species, taxa or group of organisms and, if required, life stage.
Metabolic Domain	Consideration of whether the alert requires, or does not require, metabolic activation.
Mechanistic Interpretation	The structural alert is associated with a recognisable and/or understandable mechanism of toxic action, in addition to, where possible, an AOP.
Mechanistic Causality	The definition of the structural alert in terms of structural chemistry, physico-chemical properties etc., is related to the MIE or KE of the mechanism/AOP in a comprehensible/plausible fashion. If possible, the structural alert should relate to the mechanism of action in terms of the chemistry that underpins the interaction with physiological/biochemical processes. E.g. a structural alert for covalent DNA binding should be related to an organic chemistry reactive mechanism. It is noted that an alert may be mechanistically interpretable, but lack mechanistic causality.
Coverage	The coverage is the relative proportion of hits a structural alert would have within a defined chemical inventory.
Performance	The performance of a structural alert can be defined in terms of its predictivity, or ability to match compounds known to be associated with that effect. Ideally structural alerts will have a good prediction rate for positives, and low false positive prediction rate. However, this is dependent, in part at least, on the purpose of the structural alert i.e., toxicity prediction versus grouping or screening.
Corroborating Evidence	The availability of source toxicological, effect or other data that support, or were used to create, the structural alert, e.g., that it may be directly relevant to a toxicological endpoint, adverse effect, MIE etc.
Supporting Evidence	The availability of additional information that may support a weight of evidence approach e.g. data from omics or *in vitro* assays, or data from other endpoints or non-standard tests, that support the structural alert and provide evidence for the mechanism of action or related to an AOP, but which may not have been considered in the development of the alert. Direct mechanistic relevance may be difficult for many endpoints.

The first criterion (as stated in Table 2) for the assessment of structural alerts relates to its “Purpose” which will ensure that a proper use case scenario has been assigned. The following five criteria (Structural Description, Property Domain, Toxicity or Relationship to Adversity, Species Specificity, Metabolic Domain) attempt to define uncertainty associated with the definition of the alert and its applicability. It is essential that a structural alert must be adequately defined in terms of chemical structure or toxicophore, otherwise it will be difficult or impossible to use. Its description should be explicit and ideally comprise any confounding or influencing factors e.g., that may promote a change, increase or decrease in activity. It is important to note that slight differences in structure may be associated with large changes in activity and toxic effects, this is often termed an “activity cliff” (Maggiora, 2006). Such minor differences in structure may affect reactivity, and hence endpoints such as skin sensitisation (Pestana et al., 2022) or receptor binding, notable for reproductive effects (Mori et al., 2018). To be accurate, structural alerts must encode this information to avoid over-prediction. The definition of the domain of alerts is assisted by consideration of all data, for instance *in chemico* data have been utilised to define the domains of a number of reactive mechanisms associated with skin sensitisation (Richarz et al., 2014; Rodriguez-Sanchez et al. (2013); Nelms et al. (2013)).

The definition of the domain associated with physico-chemical properties will allow for cut-offs, e.g., for solubility or volatility, to be incorporated which will account, in part at least, for elements of toxicokinetics. At the current time, this aspect of the domain is seldom characterised. However, a broad (or no) physico-chemical property domain will extend the coverage of an alert, and strict cut-offs will restrict coverage, i.e., general or highly specific respectively. The definition of domain in terms of physico-chemical properties must implicitly be derived from training set data and hence is likely to forge a link with species specificity. In most cases, physico-chemical properties are likely to be related to the toxicokinetics of a compound, i.e. an alert may indicate the toxicodynamic possibility of initiating toxicity, but this may be tempered by adverse toxicokinetic properties. The incorporation of a physico-chemical property and/or descriptor domain may ultimately allow for some form of quantification, as demonstrated recently with regard to determining groupings of potency for repeated dose toxicity (Yang et al., 2021), increasing reactivity or bioavailability that may be associated with skin sensitisation (Natsch et al., 2015) or the Cramer Classes for systemic toxicity (Cramer et al., 1978).). Alternatively, structural alerts without physico-chemical properties can be used in combination with QSAR models, where the structural alert guides the user to select the appropriate QSAR model that relates to the mechanism predicted by the structural alert (e.g. a QSAR model for non-polar narcosis), while the QSAR itself incorporates the physico-chemical properties allowing for quantitative prediction. The toxicity, endpoint or adverse effect predicted should be defined, along with the species to which it is relevant. With regard to definition of species, this will be dependent on the training set and endpoint. There are also examples, e.g., alerts for the inhibition of acetylcholinesterase, where the alert will be very broadly applicable, and even a statement such as “any species with acetylcholinesterase” may be seen as appropriate. The species applicability of some alerts may also be defined by extrapolation, using for instance protein orthology databases (LaLone et al., 2016), which is, of course, leading to higher uncertainty, but at the same time greatly enhancing the species applicability domain of the alert. In terms of metabolism, it is acknowledged that some alerts implicitly imply metabolism and this is captured in the description of the alert. An example being for the DNA reactivity of an aromatic amine, which implicitly includes a metabolic step to the nitrenium ion or nitroso derivative (Bauer et al., 2018; Enoch and Cronin, 2010). However, not all metabolic transformations are captured implicitly in alerts, with some requiring knowledge of metabolism or use of a metabolic simulator with the alert only being observed in the metabolite e.g. some phenols can be oxidised to the corresponding quinone which may be a skin sensitiser, whilst the alert is often associated with the quinone alone (Bajot et al., 2011). There are also many direct acting, non-metabolically activated, alerts for toxicity. The purpose of this criterion is that the requirement (or not) for metabolism should be stated, or if this knowledge is not known it should be acknowledged as an uncertainty. The uncertainty is not in the requirement for metabolism, but whether it is known and stated unambiguously.

The evidence of causality of an alert i.e., that it is plausible, is captured partially by mechanistic relevance with two criteria (Mechanistic Interpretation and Mechanistic Causality) and related to the criteria describing the availability of corroborating or supporting evidence. Mechanistic relevance is important to provide evidence of causality, i.e., that it is toxicologically meaningful, and hence the transparency of an alert. In this case Mechanistic Interpretation ascertains the confidence in there being a recognisable mechanism of action that can be associated to the SAR and, ultimately, structural alert. Mechanistic Causality is whether the description of the structural alert, in terms of chemistry or properties, is related to the mechanism of action. Reference to AOPs is highly useful in this context (OECD, 2017), particularly with regard to MIEs which may drive structural alerts (Cronin and Richarz, 2017). The two criteria are not independent and assessing Mechanistic Causality is not possible without knowledge of Mechanistic Interpretation, or at very least knowledge of a potential mechanism and/or MIE. This is important to demonstrate the veracity of an alert, although it is acknowledged that full mechanistic interpretation may not be possible for all alerts i.e., when the mechanisms are unknown or debated. Supporting evidence is addressed with two criteria. Corroborating Evidence relates to relevant biological data, e.g., *in vivo* assays, or *in vitro* data relating and confirming a mechanism or adverse outcome directly, that support the structural alert. Corroborating Evidence can also relate to high-throughput or high content data, for instance to explore alerts associated with MIEs and KEs (Wang et al., 2019, 2021). Supporting Evidence is other evidence or data streams, which may have lower levels of biological complexity, e.g., other *in vitro* data, high content screening, omics outputs etc., that support weight of evidence to provide the mechanistic relevance of the structural alert. Supporting Evidence can, however, also include other information such as data from related endpoints, non-standard data etc., for instance the use of mutagenicity data to support the assessment of skin sensitisation (Mekenyan et al., 2010).

The final two criteria to consider (Coverage, Performance) are objective and will assist in understanding how an alert can be used. Coverage can be defined as the number of hits the alert has in a chemically diverse database featuring the alert; this is, of course, reliant on the nature of a database and is relative only to that and for a specific alert. It will give information on whether an alert is general in nature i.e., high coverage, or specific, i.e. low coverage. Performance can be assessed with a number of statistical criteria, e.g. Cooper statistics (Cooper et al., 1979), Fisher's exact test (as exemplified in Wang et al., 2019); it is noted that there are few alerts associated with the absence of a given mechanism of toxicity – although they could, for instance, be derived from machine learning – and the “negatives” in Cooper statistics should only be considered when there is a negative alert, but should not be considered for positive alerts with the absence of an alert analogous to a negative outcome, hence prediction of “negatives”, i.e. non-toxic molecules, should be ignored in this situation. Dependent on the use of the alert, some scenarios, e.g., low false negative rate, may be preferred. With particular reference to data-driven methods of determining structural alerts, there may be a need to consider the use of test and training sets to assess the performance and significance of an alert if there is no underlying expert knowledge at the outset, similar to the development of other types of *in silico* models. It is obvious that such statistics are dependent on the quality and extent of any underlying data set as well as how strictly the alert is defined both in terms of chemical structure and physico-chemical properties. As such, these criteria should not be considered to exclude alerts, but will provide an estimate of the confidence provided by associated data i.e. if there are few data to support and alert, it may indicate that further data should be sought.

### 3.2 Scheme to assign a confidence score to a structural alert

A key component of the scheme to define uncertainty was the possibility of investigating the (semi-) quantitative assignment of confidence to an alert. To achieve this, the twelve assessable criteria were defined in terms of low, moderate or high confidence, as reported in Table 3. From the outset, it is important to state that low confidence in one or more criteria may be acceptable under certain circumstances. The purpose, in line with the ethos applied by EFSA (2018) with regard to uncertainty, is to highlight areas where improvement in confidence may be achievable to improve the acceptability of a prediction involving a structural alert for a specific purpose. The scheme will also allow for the comparison of the reliability of alerts within, for instance, weight of evidence approaches. Whilst the current scheme assigns confidence into one of three classes i.e., low, moderate and high, it is acknowledged that more classifications could be assigned. An analysis of the advantages and disadvantages of including more classifications of uncertainty is provided by Cronin et al. (2019) with regard to the assessment of the uncertainty of QSARs.

**Table 3:** Definitions of the properties relating to structural alerts and their relevance to confidence.

Criterion	Confidence	Relevance to the Structural Alert in Terms of Possible Uncertainty Affecting Confidence
Purpose	High	The purpose of the structural alert is clearly and unambiguously stated, e.g., toxicity prediction or grouping.
	Moderate	The purpose of the structural alert is broad or ambiguous.
	Low	The purpose of the structural alert is not stated.
Structural Description	High	Unambiguous description of the functional group and/or molecular fragment including modulating factors.
	Moderate	Structural alert is loosely defined with regard to its chemical structure with little or no information regarding modulating factors.
	Low	Poor, or no, description of the structural alert with regard to its chemical structure or modulating factors.
Property Domain	High	A well-defined domain in terms of the complete molecular environment and ranges of physico-chemical and/or structural properties.
	Moderate	Some, but incomplete, definition of the domain for the complete molecular environment. No, or incomplete, definition of the ranges of physico-chemical and/or structural properties.
	Low	No, or very ambiguous, definition of the domain for the complete molecular environment and the ranges of physico-chemical and/or structural properties.
Toxicity or Relationship to Adversity	High	The endpoint, toxicity or adverse effect(s) is clearly and unambiguously stated.
	Moderate	The endpoint, toxicity or adverse effect(s) is general and lacks specificity e.g. in terms of organ or species.
	Low	The endpoint, toxicity or adverse effect(s) is not known or stated.
Species Specificity	High	The species, taxa or groups of organisms, in addition to relevant life stage if important, to which the structural alert is relevant, are identified and clearly stated.
	Moderate	There is some evidence and documentation that the structural alert is associated with the species to which it pertains.
	Low	No evidence is presented for a species-specific response to the structural alert.
Metabolic Domain	High	The metabolic domain is clearly and unambiguously stated e.g., the alert defines whether a chemical does or does not require metabolic activation.
	Moderate	The metabolic domain is ambiguous or poorly defined.
	Low	The metabolic domain is not known or stated.
Mechanistic Interpretation	High	The structural alert is strongly associated with a well-recognised and documented mechanism of action, e.g., a well-developed or OECD endorsed AOP.
	Moderate	The structural alert is possibly associated with a mechanism of action.
	Low	There is no mechanism of action or no documentation associated with the structural alert.
Mechanistic Causality	High	The chemistry captured by the structural alert is strongly associated with the MIE and/or a KE of the mechanism of action.
	Moderate	There is possible, but unsubstantiated, evidence that the chemistry of the structure may be associated with the mechanism of action, for instance evidence of correlation but not causality.
	Low	The chemistry captured by the structure alert has no documented association with the mechanism of action.
Coverage	High	The structural alert has relatively low coverage of alert-specific chemical space which could imply a limited and well-defined domain.
	Moderate	The structural alert has general coverage of alert-specific chemical space with a moderately broad domain.
	Low	The structural alert has high, or undefined, coverage of alert-specific chemical space indicating a broad, unspecific alert.
Performance	High	A statement relating to the predictive performance of the structural alert to assist in understanding the purpose of the alert, i.e., good performance measured by few false positives/negatives for hazard identification, or biased to ensure few false negatives for screening in a tiered approach.
	Moderate	The structural alert has modest (i.e. greater than random but is not 100% accurate) predictive performance.
	Low	The structural alert is not able to distinguish between active and inactive chemicals.
Corroborating Evidence	High	Multiple and confirmatory toxicological data to support the structural alert.
	Moderate	Few toxicological data exist to support the structural alert.
	Low	No toxicological data are available to support the structural alert e.g. for a statistical approach or one derived on hypothetic mechanisms.
Supporting Evidence	High	Multiple and confirmatory evidence from mechanistic information to confirm the mechanistic hypothesis.
	Moderate	Few data exist to support the mechanistic interpretation of the structural alert.
	Low	No mechanistic information is available to support the structural alert.

The criteria for assessment of structural alerts have different levels of relevance for a given purpose. Table 4 provides a putative evaluation and ranking of the criteria with regard to their use, for instance, for hazard assessment where a point of departure may, or may not, be required. Some criteria, e.g., the definition of the alert, are essential to the use of an alert. Others could have lower confidence, especially with regard to evidence for causality i.e., mechanistic and metabolic understanding. Whilst mechanistic understanding is desirable, the absence of complete mechanistic understanding should not preclude the use of an established and plausible structural alert. A similar argument can be made of metabolic understanding – i.e., in many cases this may be obvious or can be implied, but lack of complete knowledge of how metabolism may affect an alert should not preclude its use. In addition, metabolic competency may be species dependent and may activate or inactivate a MIE. A further set of criteria, mainly related to the properties and supporting information of the alert, are considered less critical for the evaluation of confidence.

**Table 4:** Proposed relative importance of the confidence criteria in the scheme for the assessment of structural alerts relating to acceptable levels of confidence. In this case, the attributes are for hazard identification supporting risk assessment.

Criteria	Comment
*Essential Attributes of a Structural Alert – Must be Associated with High Confidence (where possible)*	
Structural Description	The alert must be explicitly defined in terms of its chemical structure, structural domain and which species it is relevant to.
Property Domain	
Toxicity or Relationship to Adversity	
Species Specificity	
Corroborating Evidence	
*Desirable Attributes of a Structural Alert – Preferably Associated with High Confidence (where possible)*	
Metabolic Domain	The mechanistic and metabolic relevance of an alert increases its transparency and potential acceptance.
Mechanistic Interpretation	
Mechanistic Causality	
*Optional Attributes of a Structural Alerts – Where Possible Associated with High/Moderate Confidence*	
Purpose	Statistical analysis and source data increase the credibility, or otherwise, of a structural alert.
Coverage	
Performance	
Supporting Evidence	

The application of the criteria and relative assessment of confidence is provided for three different types of alerts in the Supplementary Information Tables S1–S3 respectively. The structural alerts considered are for aliphatic alcohols with reference to acute toxicity across multiple environmental species (taken from Sapounidou et al., 2021 and analogous to that from Verhaar et al., 1992), the ability of aromatic amines to bind to DNA (Enoch and Cronin, 2010) and the inhibition of acetylcholinesterase (AChE) by 1-indanone (Figure 2 in Wedlake et al. (2020)). There are significant differences between these alerts in that those for the aliphatic alcohol and aromatic amine moieties are based on considerable expert knowledge and are well-supported by experimental data. The alert for AChE inhibition is data-driven being derived from data from *in vitro* assays. The differences are reflected in the scores. For instance, Tables S1 and S2 indicate both the alcohol and amine alerts are well defined with a strong mechanistic background. However, low confidence was apparent in the lack of information on coverage and performance. As both alerts are intended for grouping, rather than direct toxicity prediction, this may be deemed acceptable if used appropriately. Table S3 indicates the alert for AChE inhibition has less direct toxicological relevance but is well characterised in terms of coverage and performance.

**Fig. 1:**
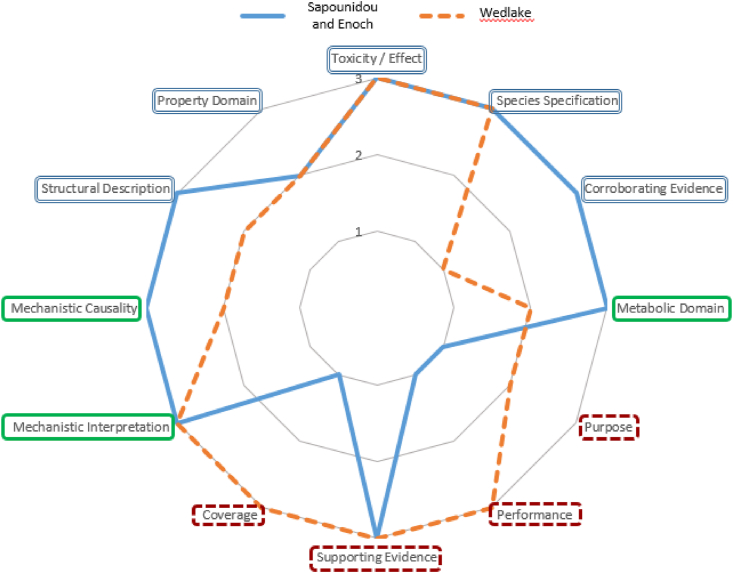
Radar plot representing the “confidence profile” associated with knowledge driven alerts (from Sapounidou and Enoch) in blue as compared to the data-driven alert (from Wedlake) in orange (dashed line). The confidence criteria are ordered according to the relative importance as stated in Table 4, with the essential criteria at the top of the radar plot in blue boxes (double line), moderately importantly in green boxes (single line) at the centre of the plot and lower importance at the base of the plot in red boxes (dashed line). The criteria have been scored from 3 (low uncertainty/high confidence) to 1 (high uncertainty/low confidence). (For interpretation of the references to colour in this figure legend, the reader is referred to the Web version of this article.)

The relative confidence that can be associated with the three structural alerts is demonstrated graphically as “radar plots” in Fig. 1. The two alerts based on expert knowledge (from Sapounidou et al. (2021) and Enoch et al. (2011)) have the same “confidence profile” as defined by the criteria, with low confidence associated with the lack of documented coverage and performance of these alerts. The data-driven alert from Wedlake et al. has a different confidence profile, with lower confidence associated with the lack of primary data anchored to the alert.

Given the possibility of ranking the relative criteria for the assessment of the confidence for structural alerts according to their relevance and importance as shown in Table 4, it may also be possible to allocate some type of weighting to create a score for a particular alert that takes account of the particular levels of confidence. This can be converted to give a “confidence score” for a particular alert. A proposed scheme to add a weighting to each of the criteria is given in Supplementary Information Tables S1–S3 and has been applied to the three alerts. For clarity, the weightings in Tables S1–S3 correspond to Table 4 i.e. essential criteria are given a weighting of 10, desirable criteria a weighting of 5 and optional criteria a weighting of 2. At this time the weightings are arbitrary and any uptake of such weighting will require consideration with regard to their use and purpose. For instance, it is anticipated that alerts could be aligned with different characteristics for hazard identification (where a highly specific, data rich alert may be required) as opposed to prioritisation and screening (where a broader alert, not necessarily mechanistically-based, may be acceptable). As such, not only different weightings, but different (semi-quantitative) weights could be applied. Where a rapid screening tool is required, for instance for the evaluation of a chemical inventory, then the most relevant characteristics of alerts will be coverage and an understanding of the false prediction rate (particularly the possibility of not identifying particular effects). To assign compounds to a particular QSAR, as in the Sapounidou et al. (2021) scheme, then much greater emphasis will be placed on the mechanistic understanding, or the relevance to the known molecular initiating events.

The weightings in the scheme are on a scale up to 10 with the higher weighting being associated with those criteria deemed more essential in Table 4. Such an analysis has the effect of emphasising the important uncertainties associated with an alert. The weighting has had the effect of emphasising the relatively low confidence (or high uncertainty) that is associated with the alert from Wedlake with regard to its structural description and the lack of primary (*in vivo*) supporting evidence. This, of course, does not preclude the use of this alert, but demonstrates where further information and/or knowledge could be provided to increase confidence in its use. In addition, there may be possibilities for using alerts not supported by *in vivo* data for specific purposes, such as to confirm an MIE. The essentiality of some criteria will depend on the use case, as noted above, and it is unlikely that a single list covering all use cases can be developed.

The Mean Confidence Score and a “Weighted Confidence Score” are also provided for the three alerts in Tables S1–S3. The Weighted Confidence Score is calculated as:

(1) $$\text{Weighted Confidence Score }=\frac{\sum \text{weighted confidence scores for each criterion}}{\sum \text{weightings for each criterion}}$$

The resulting scores are on a scale from 3 (greatest confidence) to 1 (lowest confidence). Having a single number for a Confidence Score is in some ways appealing i.e., a number can provide information on confidence, but runs a very high risk of being misleading if misinterpreted. It is not intended that a higher score implies any alert to be “better” than any other alert, but that it may be better defined in certain characteristics which could make it more amenable for various use cases. The Confidence Scores for the knowledge-based alerts (Sapounidou and Enoch) are higher than for the data-driven alert, however this does not take account of other factors such as speed of development. It should also be emphasised that a single score for confidence may mask an unacceptable uncertainty in one, or a small number, of areas. Thus, close examination of radar plots, such as Fig. 1, is helpful and inevitably leads to the question of what the desirable characteristics of an alert for a specific purpose are, which is considered in the next section. Since weightings in any scheme are defined by the user, they can be adjusted to emphasise any particular aspect of the evaluation.

### 3.3 Use cases and the desired properties for structural alerts

Five use case scenarios for structural alerts are described below, with attributes noted in Table 5. These do not encompass all uses, but are representative of the types of applications for which structural alerts may be used. These include those for regulatory use and industry specific uses, namely:

• Hazard identification through direct prediction of toxicity to support risk assessment, e.g., giving weight to a particular adverse outcome.
• Mechanism-based analogue identification, e.g., to select similar compounds or analogues as part of a read-across to enable mechanistic justification, assignment of a particular chemical to a QSAR, such as a reactive or specific mechanism.
• Category identification e.g., assigning a compound as a chemical class-based analogue for High Production Volume (HPV) chemicals.
• Predictions of effects, or identification of hazard, leading to classification and labelling in a regulatory context.
• Screening and/or prioritisation e.g., to identify or highlight potentially hazardous compounds in a regulatory context or as part of product development.

**Table 5:** Ideal levels of confidence and characteristics of structural alerts in different use case scenarios. The ideal levels of confidence are defined in Table 3.

Criteria	Hazard Identification Supporting Risk Assessment	Mechanism-Based Analogue Identification	Category Identification e.g., Chemical Class-Based Analogue for HPV Chemicals	Predictions Leading to Classification and Labelling	Screening and/or Prioritisation
Structural Description	High	High	High	High	High
Property Domain	High	High	High	Moderate	Moderate
Toxicity or Relationship to Adversity	High	High	High	High	High
Species Specificity	High	High	High	High	Moderate
Metabolic Domain	High/Moderate	High/Moderate	High/Moderate	Moderate	Moderate
Purpose	High	High	Moderate	Moderate	Moderate
Mechanistic Interpretation	High	High	High	High	High
Mechanistic Causality	High	High	Moderate/High	Moderate/High	Moderate/High
Coverage	High	High	Low	Low	Low
Performance	High	High	Moderate	Moderate	Moderate
Corroborating Evidence	High	High	Moderate	Moderate	Moderate
Supporting Evidence	Moderate	Moderate	Moderate/Low	Low	Low

Table 5 provides an estimate of the ideal minimum levels of confidence that might be required for each of the twelve uncertainty criteria. From the outset, it is clear that different levels of confidence are acceptable for different use case scenarios. Those associated with providing input into hazard identification, i.e., direct prediction of toxicity and read-across, ideally have higher confidence. Lower confidence may be acceptable for screening and prioritisation.

Some characteristics of structural alerts should be definitive regardless of use case and hence be associated with high confidence. Examples of these include the structural description of the fragment, the endpoints to which it relates and the species relevance. In addition, the definition and understanding of confidence in structural alerts suggests that different use case scenarios could potentially utilise different characteristics of structural alerts. The identification of analogues, for instance as a primary categorisation tool for read-across, requires highly defined structural alerts with good mechanistic understanding. The purpose here is to identify very closely related chemicals as defined by their structural alerts that would support a robust argument for similarity. The use of structural alerts for assignment of compounds to chemical classes could have lower confidence in terms of structural definition. This would allow for a larger number of compounds to be grouped together, and associated with this could be lower mechanistic understanding with the possible expectation of sub-categorisation later on to allow for efficient analogue selection. However, for uses such as hazard identification or prioritisation, lower confidence may be acceptable to allow for the identification of potential toxicants, with the possibility of false positives being ameliorated by further evidence or testing.

Knowledge from the scheme for the evaluation of confidence of structural alerts can also help indicate how to use alerts. For instance, the aliphatic alcohol and aromatic amine alerts (assessed in Tables S1 and S2 respectively) are associated with low confidence for their coverage and performance, as these statistics are not known. In addition, they can be considered as quite broadly defined, thus likely to capture or identify many analogues in a read-across scenario. In such a situation sub-categorisation is recommended, for instance using similarity indices (Mellor et al., 2019). Thus, the evaluation of confidence through the scheme presented does not preclude the use of any structural alert but will assist in the identification of how and where they can be used optimally and justifiably. Other aspects to be considered are the definition of the various domains i.e., structural, mechanistic and metabolic. As noted above and in Table 4, high confidence in the structural definition is a pre-requisite for use, whilst mechanistic and, in particular, metabolic definition may be more aspirational.

## 4 Conclusions

A scheme is proposed that characterises the uncertainty associated with structural alerts in an attempt to understand the confidence that may be associated with them. Twelve criteria have been considered that account for the quality and usability of an alert for a specific purpose. These criteria have been ranked according to how essential they are for a particular use case. Assessment of existing alerts suggests that those derived directly from expert knowledge have different uncertainties to those from data-driven analyses. This does not discount any particular method of alert creation, rather these findings can be used to reduce uncertainty through finding further data and information to increase confidence in the use of these predictive approaches as well as allowing for increased confidence on decisions made on the alerts and for benchmarking existing alerts.

## Disclaimer

The views expressed in this article are those of the authors and do not necessarily reflect the views or positions of the US Environmental Protection Agency. Reference to commercial products or services does not constitute endorsement.

## Funding body information

All authors were funded via their own institutions.

## CRediT authorship contribution statement

**Mark T.D. Cronin:** Conceptualization, Investigation, Methodology, Writing – original draft, Writing – review & editing, Visualization. **Franklin J. Bauer:** Conceptualization, Methodology, Writing – original draft, Writing – review & editing. **Mark Bonnell:** Conceptualization, Methodology, Writing – original draft, Writing – review & editing. **Bruno Campos:** Conceptualization, Methodology, Writing – original draft, Writing – review & editing. **David J. Ebbrell:** Writing – review & editing. **James W. Firman:** Writing – review & editing. **Steve Gutsell:** Conceptualization, Methodology, Writing – original draft, Writing – review & editing. **Geoff Hodges:** Conceptualization, Methodology, Writing – original draft, Writing – review & editing. **Grace Patlewicz:** Conceptualization, Methodology, Writing – original draft, Writing – review & editing. **Maria Sapounidou:** Writing – review & editing. **Nicoleta Spînu:** Writing – review & editing. **Paul C. Thomas:** Conceptualization, Methodology, Writing – original draft, Writing – review & editing. **Andrew P. Worth:** Conceptualization, Methodology, Writing – original draft, Writing – review & editing.

## Declaration of competing interest

The authors declare that they have no known competing financial interests or personal relationships that could have appeared to influence the work reported in this paper.
