# Supplementary material for: A scheme to evaluate structural alerts to predict toxicity – Assessing confidence by characterising uncertainties
Source: Regul Toxicol Pharmacol. 2022 Nov;135:105249. doi: 10.1016/j.yrtph.2022.105249 (PMC9585125; doi:10.1016/j.yrtph.2022.105249)
Supplement: Multimedia component 1 [file mmc1.docx]

**A Scheme to Evaluate Structural Alerts to Predict Toxicity – Assessing Confidence By Characterising Uncertainties**

Mark T.D. Cronin, Franklin J. Bauer, Mark Bonnell, Bruno Campos, David J. Ebbrell, James W. Firman, Steve Gutsell, Geoff Hodges, Grace Patlewicz, Maria Sapounidou, Nicoleta Spînu, Paul C. Thomas, Andrew P Worth

**Supplementary Information**

Table S1. Application of the weighting scheme for evaluation of the confidence of structural alerts for aliphatic alcohols with reference to acute fish lethality (taken from Sapounidou et al., 2021).

| Alert: | **Aliphatic alcohols, but NOT allylic/propargylic alcohols** | | | | | |
| --- | --- | --- | --- | --- | --- | --- |
| *Purpose identification* | Classification: Integral part of Sapounidou’s MOA Classification Scheme | | | | | |
|  | | | | | | |
| **Criteria** | **Confidence** | **Comment / Justification** |  | Confidence Score | Weighting | Weighted Confidence |
|  |  |  |  |  |  |  |
| Purpose | High - The purpose of the structural alert is clearly and unambiguously stated e.g., toxicity prediction or grouping. | Integral part of Sapounidou Scheme |  | 3 | 2 | 6 |
| Structural Description | High - Unambiguous description of the functional group and / or molecular fragment including modulating factors. | Well defined |  | 3 | 10 | 30 |
| Property Domain | Moderate - Some, but incomplete, definition of the domain for the complete molecular environment. No, or incomplete, definition of the ranges of physico-chemical and / or structural properties. | Descriptors ranges not known |  | 2 | 10 | 20 |
| Toxicity or Relationship to Adversity | High - The endpoint, toxicity or adverse effect is clearly and unambiguously stated. | Fish acute toxicity |  | 3 | 10 | 30 |
| Species Specificity | High - The species, taxa or groups of organisms to which the structural alert is relevant are identified and clearly stated. | Guppy and fish in general |  | 3 | 10 | 30 |
| Metabolic Domain | High - The metabolic domain is clearly and unambiguously stated e.g., the alert defines whether a chemical does or does not require metabolic activation. | No metabolism |  | 3 | 5 | 15 |
| Mechanistic Interpretation | High - The structural alert is strongly associated with a well recognised and documented mechanism of toxic action, e.g., an AOP. | Associated with Non-Polar Narcosis |  | 3 | 5 | 15 |
| Mechanistic Causality | High – The chemistry captured by the structure alert is strongly associated with the MIE and / or KE of the mechanism of action. | Associated with non-reactive chemicals |  | 3 | 5 | 15 |
| Coverage | Low – Coverage not known |  |  | 1 | 2 | 2 |
| Performance | Low – Performance not known |  |  | 1 | 2 | 2 |
| Corroborating Evidence | High – There are significant toxicological data to support the structural alert. | Many toxicological data are available to support the alert |  | 3 | 10 | 30 |
| Supporting Evidence | High – There is significant evidence from mechanistic information to confirm the mechanistic hypothesis. | Considerable mechanistic evidence is available |  | 3 | 2 | 6 |
|  |  |  |  |  |  |  |
|  |  |  |  | TOTAL | 73 | 201 |
|  |  |  |  |  |  |  |
|  |  | **Mean Confidence Score** | **2.58** |  |  |  |
|  |  | **Weighted Confidence Score (201/73)** | **2.75** |  |  |  |
|  |  |  |  |  |  |  |

Table S2. Application of the weighting scheme for evaluation of the confidence of structural alerts for ability of aromatic amines to bind to DNA (Enoch and Cronin, 2010).

| Alert: | **Aromatic Amine** | | | | | |
| --- | --- | --- | --- | --- | --- | --- |
| *Purpose identification* | Classification: Integral part of DNA Binding Alerts profiler and previously in OECD QSAR Toolbox | | | | | |
|  | | | | | | |
| **Criteria** | **Confidence** | **Comment / Justification** |  | Confidence Score | Weighting | Weighted Confidence |
|  |  |  |  |  |  |  |
| Purpose | High – The purpose of the structural alert is clearly and unambiguously stated e.g., toxicity prediction or grouping. | Integral part of an *in silico* profiler designed for grouping and read-across e.g., through the OECD QSAR Toolbox |  | 3 | 2 | 6 |
| Structural Description | High - Unambiguous description of the functional group and / or molecular fragment including modulating factors. | Well defined |  | 3 | 10 | 30 |
| Property Domain | Moderate - Some, but incomplete, definition of the domain for the complete molecular environment. No, or incomplete, definition of the ranges of physico-chemical and / or structural properties. | Descriptors ranges not known, but mitigating fragments, etc. defined |  | 2 | 10 | 20 |
| Toxicity or Relationship to Adversity | High - The endpoint, toxicity or adverse effect is clearly and unambiguously stated. | Covalent binding to DNA that may ultimately cause disruption of DNA and lead to genotoxicity |  | 3 | 10 | 30 |
| Species Specificity | High - The species, taxa or groups of organisms to which the structural alert is relevant are identified and clearly stated. | Species with DNA may be susceptible |  | 3 | 10 | 30 |
| Metabolic Domain | High - The metabolic domain is clearly and unambiguously stated e.g., the alert defines whether a chemical does or does not require metabolic activation. | Metabolism is defined as part of the alert |  | 3 | 5 | 15 |
| Mechanistic Interpretation | High - The structural alert is strongly associated with a well recognised and documented mechanism of toxic action, e.g., an AOP. | MIE for genotoxicity |  | 3 | 5 | 15 |
| Mechanistic Causality | High – The chemistry captured by the structural alert is strongly associated with the MIE and / or KE of the mechanism of action. | Associated with known electrophilic mechanism |  | 3 | 5 | 15 |
| Coverage | Low – Coverage not known |  |  | 1 | 2 | 2 |
| Performance | Low – Performance not known |  |  | 1 | 2 | 2 |
| Corroborating Evidence | High – There are significant toxicological data to support the structural alert. | Many toxicological data are available to support the alert |  | 3 | 10 | 30 |
| Supporting Evidence | High – There is significant evidence from mechanistic information to confirm the mechanistic hypothesis. | Considerable mechanistic evidence is available |  | 3 | 2 | 6 |
|  |  |  |  |  |  |  |
|  |  |  |  | TOTAL | 73 | 182 |
|  |  |  |  |  |  |  |
|  |  | **Mean Confidence Score** | **2.58** |  |  |  |
|  |  | **Weighted Confidence Score (201/73)** | **2.75** |  |  |  |
|  |  |  |  |  |  |  |

Table S3. Application of the weighting scheme for evaluation of the confidence of structural alerts for the alert for 1-indanone relating to the inhibition of acetylcholinesterase (AChE) (Figure 2 in Wedlake, 2020).

| Alert: | **Alert for 1-Indanone for Inhibition of Acetylcholinesterase (AChE)** | | | | | |
| --- | --- | --- | --- | --- | --- | --- |
| *Purpose identification* | Toxicity Prediction: Data-driven alert based on knowledge of Molecular Initiating Event | | | | | |
| **Criteria** | **Confidence** | **Comment / Justification** |  | Confidence Score | Weighting | Weighted Confidence |
|  |  |  |  |  |  |  |
| Purpose | Moderate – The purpose of the structural alert is broad or ambiguous. | One of many alerts in a suite of alerts derived from toxicologically relevant MIEs |  | 2 | 2 | 4 |
| Structural Domain | Moderate – Structural alert is loosely defined with regard to its chemical structure with little or no information regarding modulating factors. | Modulating groups not defined although could be assumed from the training set, |  | 2 | 10 | 20 |
| Property Domain | Moderate – No, or incomplete, definition of the ranges of physico-chemical and / or structural properties. | Descriptors ranges not known. |  | 2 | 10 | 20 |
| Toxicity or Relationship to Adversity | High – The endpoint, toxicity or adverse effect is clearly and unambiguously stated. | Inhibition of AChE leading to neurotoxicity. |  | 3 | 10 | 30 |
| Species Specificity | High – The species, taxa or groups of organisms to which the structural alert is relevant are identified and clearly stated. | Assumed to be any species with AChE |  | 3 | 10 | 30 |
| Metabolic Domain | Moderate – The metabolic domain is ambiguous or poorly defined. | Metabolism is not defined as part of the alert, however it is probable (though not stated) that there is no metabolic activation |  | 2 | 5 | 10 |
| Mechanistic Interpretation | High – The structural alert is strongly associated with a well recognised and documented mechanism of toxic action, e.g. an AOP. | MIE for inhibition of AChE |  | 3 | 5 | 15 |
| Mechanistic Causality | Moderate – There is possible, but undocumented, evidence that the chemistry of the structure there may be associated with the mechanism of action. | The basis for the interaction with AChE is not stated |  | 2 | 5 | 10 |
| Coverage | High – The structural alert has relatively low coverage of chemical space with few false positives. | Well defined coverage |  | 3 | 2 | 6 |
| Performance | High – The structural alert has excellent predictive performance, i.e., specificity and sensitivity. | Excellent performance as demonstrated by predictivity of training / test sets |  | 3 | 2 | 6 |
| Corroborating Evidence | Low - No toxicological data are available to support the structural alert. | No toxicological data are provided to support the alert |  | 1 | 10 | 10 |
| Supporting Evidence | High - There is significant evidence from mechanistic information to confirm the mechanistic hypothesis. | Mechanistic information from the MIE and data relating to training / test sets |  | 3 | 2 | 6 |
|  |  |  |  |  |  |  |
|  |  |  |  | TOTAL | 73 | 167 |
|  |  |  |  |  |  |  |
|  |  | **Mean Confidence Score** | **2.42** |  |  |  |
|  |  | **Weighted Confidence Score (167/73)** | **2.28** |  |  |  |
|  |  |  |  |  |  |  |
